# Isolation and Characterization of Polysaccharides from the Ascidian *Styela clava*

**DOI:** 10.3390/polym14010016

**Published:** 2021-12-22

**Authors:** Jesus Valcarcel, José Antonio Vázquez, Uxía R. Varela, Rui L. Reis, Ramon Novoa-Carballal

**Affiliations:** 1 Recycling and Valorisation of Waste Materials, Marine Research Institute (IIM-CSIC), Eduardo Cabello 6, 36208 Vigo, Spain; jvazquez@iim.csic.es (J.A.V.); uxiram@hotmail.com (U.R.V.); 2 3B’s Research Group—Biomaterials, Biodegradables and Biomimetics, University of Minho, Headquarters of the European Institute of Excellence on Tissue Engineering and Regenerative Medicine, AvePark, Barco, 4805-017 Guimaraes, Braga, Portugal; rgreis@i3bs.uminho.pt; 3 ICVS/3B’s—PT Government Associate Laboratory, 4805-017 Guimaraes, Braga, Portugal

**Keywords:** *Styela clava*, ascidian, cellulose, polysaccharides, tunicate, glycosaminoglycans, valorization

## Abstract

*Styela clava* is an edible sea squirt farmed in Korea that has gradually invaded other seas, negatively impacting the ecology and economy of coastal areas. Extracts from *S. clava* have shown wide bioactivities, and ascidians have the unique capability among animals of biosynthesizing cellulose. Thus, *S. clava* is a relevant candidate for valorization. Herein, we aimed at surveying and characterizing polysaccharides in both tunic and flesh of this ascidian. To this end, we enzymatically hydrolyzed both tissues, recovering crystalline cellulose from the tunic with high aspect ratios, based on results from microscopy, X-ray diffraction, and infrared spectroscopy analyses. Alkaline hydroalcoholic precipitation was applied to isolate the polysaccharide fraction that was characterized by gel permeation chromatography (with light scattering detection) and NMR. These techniques allowed the identification of glycogen in the flesh with an estimated Mw of 7 MDa. Tunic polysaccharides consisted of two fractions of different Mw. Application of Diffusion-Ordered NMR allowed spectroscopically separating the low-molecular-weight fraction to analyze the major component of an estimated Mw of 40–66 kDa. We identified six different sugar residues, although its complexity prevented the determination of the complete structure and connectivities of the residues. The two more abundant residues were N-acetylated and possibly components of the glycosaminoglycan-like (GAG-like) family, showing the remaining similarities to sulfated galactans. Therefore, *Styela clava* appears as a source of nanocrystalline cellulose and GAG-like polysaccharides.

## 1. Introduction

*Styela clava*, a member of the ascidian class of tunicates, has become in the last decades a widespread invasive species, disrupting the ecological balance of some coastal areas and causing losses in the aquaculture industry [1,2]. Probably aided by human activities, *S. clava* overcame its native NW Pacific boundaries to first colonize California in the 1920s and Europe in the 1950s, and subsequently spread to other North American regions, Australia, New Zealand, and the Mediterranean [3]. Nevertheless, *S. clava* is farmed in Korea, considered a delicacy, and, curiously, exported to Europe and North America [4].

The edible insides of *S. clava* are enclosed in a protective, rough tunic characteristic of ascidians, which is generally regarded as waste material. Unlike any other animal, ascidians build this sheltering structure based on cellulose, presenting remarkable features, as the tunic consists of highly crystalline cellulosic nanofibers [5]. Such structures possess large surface areas, high mechanical strength, chemical resistance, show biocompatibility, and come from renewable sources, making them attractive as new materials for many applications [6]. Specifically for *S. clava*, nanocellulose isolated from the ascidian has been dispersed in organic solvents, facilitating the formulation of nanocomposites with reinforced mechanical properties [5]. In the biomedical field, the purified tunic of *S. clava* has shown osteoconductive effects on injured bone [7], and cellulose powder, applied in liquid form or as a membrane, has resulted in accelerated wound healing [8,9]. The isolation of cellulose nanocrystals generally involves acid hydrolysis to remove amorphous regions. In this process, treatment with sulfuric acid also leads to surface functionalization, resulting in ascidians in long and highly crystalline whiskers, capable of forming stable suspensions [10]. Considering the increasing availability of *S. clava*, its tunic may represent a valuable source of nanocellulose. Furthermore, *S. clava* collection in non-native regions might reduce its negative impact on ecosystems and help in ecological recovery.

Besides nanocellulose, *S. clava* contains other valuable compounds responsible for a range of observed bioactivities. In mice, ethanolic extracts of *S. clava* tunics have been shown to protect the skin from UV radiation [11] and the liver from hepatic injury [12], and aqueous extracts have displayed anti-inflammatory effects [13]. Enzymatic hydrolysates from *S. clava* have produced anti-inflammatory effects in zebrafish [14], in vitro antioxidant activity and growth inhibition of several cancer cell lines [15], and antihypertensive effects in patients with type 2 diabetes and hypertension [16]. Joint action on hypertension, glucose metabolism, and antioxidant activity has been ascribed to specific peptides isolated from enzymatic hydrolysates of *S. clava* flesh [17,18]. Furthermore, lipids recovered from *S. clava* have shown antioxidant and anti-proliferative effects in vitro [19] and sulfated alkenes’ antibacterial activity against *Bacillus subtilis* [20].

In other ascidian species, several authors have reported diverse bioactive polysaccharides, such as sulfated galactans in the tunic [21,22] and highly sulfated dermatan sulfate [23] and heparin/heparan sulfate analogues in the flesh [24]. In *S. clava*, however, no reports exist on polysaccharides from the flesh, and only one previous study has extracted glycosaminoglycans from *S. clava* tunic [25]. This investigation provided basic information about the constituent monosaccharides. The present work characterized polysaccharides in both tunic and flesh of invasive specimens of *S. clava* growing on the Galician coast (NW Spain) by a combination of Gel Permeation Chromatography (GPC) with light scattering detection, Nuclear Magnetic Resonance (NMR), X-Ray Diffraction (XRD), Fourier Transform Infrared Spectroscopy (FTIR), and Atomic Force and Scanning Electron Microscopy (AFM and SEM).

## 2. Materials and Methods

### 2.1. Raw Materials

Fourteen *Styela Clava* specimens (224 g) were collected in Ribeira (42°33′45″ N 8°59′20″ W) during spring and stored at −20 °C until processed. After defrosting, the animals were manually cleaned, gutted, and the tunics were separated from the internal organs. The tunics were defatted and decolored in a stirred acetone bath at room temperature for 24 h, repeating the procedure until no color was apparent in the extract. Moisture, organic matter, and ash were determined in triplicate in both tunics and internal organs [26], and the materials were subsequently freeze-dried.

### 2.2. Extraction and Isolation

**Figure 1:**
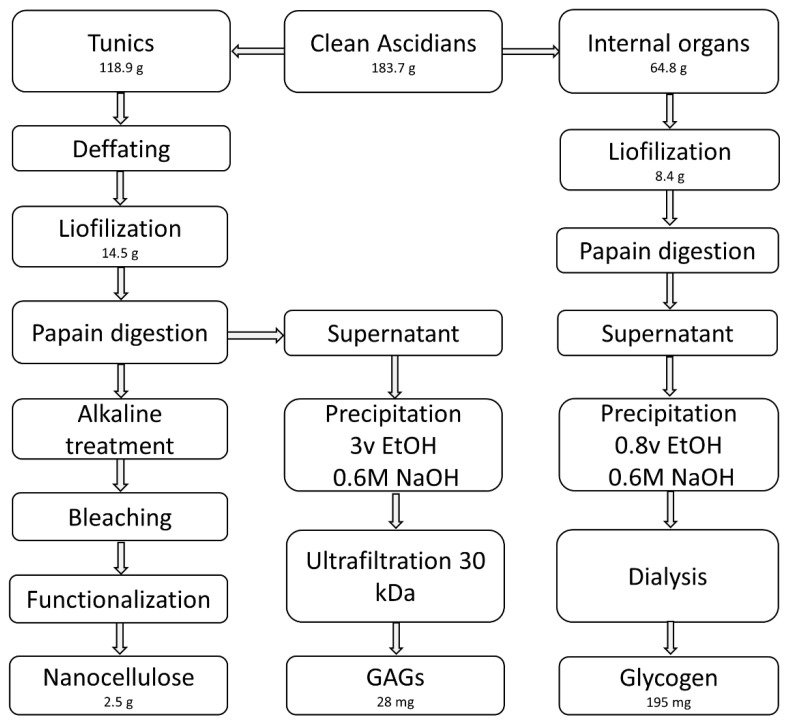
Flowchart for the isolation of polysaccharides and nanocellulose from *Styela clava*.

We performed an initial enzymatic deproteinization instead of chemical treatment to preserve the polysaccharides present in the tunic. Based on the method reported by Gomes et al. [27], 14.5 g of lyophilized tunics were stirred overnight at 60 °C in a 2-L solution containing 8 g/L of papain (2.6 U/mg, Sigma, Prod. No. P3375), 0.1 M sodium acetate (VWR, Prod. No. 27653.292), 5 mM EDTA (Sigma, Prod. No. ED2SS), and 5 mM L-cysteine (Sigma, Prod. No. 168149) adjusted to pH 5.5. Three deproteinization cycles were applied, separating the solids from the liquid fraction after each step by centrifugation (30 min at 16,783× *g*). Supernatants were combined for polysaccharide isolation, and solids also combined to obtain cellulose. The same papain digestion (once) and centrifugation were applied to the internal organs. Figure 1 shows the schematics of the isolation process.

Isolation of nanocellulose was achieved as described by van den Berg et al. [5]. The separated solids were stirred with 5% KOH (*w*/*w*) at 80 °C for 24 h and centrifuged as above specified. This procedure was repeated three times. Solids were washed with water until neutrality, followed by bleaching in a solution of 0.17% (*v*/*v*) acetic acid and 0.013% (*v*/*v*) sodium hypochlorite at 60 °C. Every hour, acetic acid and sodium hypochlorite were replenished by adding the same amounts used to prepare the initial solution, up to a total reaction time of 3 h. Then, the tunic solids were washed with water and freeze-dried, resulting in a weight of 14.5 g. The dried material was then milled in liquid nitrogen with a blender, and the resulting powder was suspended in 150 mL of water in a conical flask placed in an ice bath. Another 150 mL of sulfuric acid were slowly added (ca. 15 min), keeping the temperature inside the flask below 50 °C. Afterwards, the reaction was continued for 2 h at 45 °C in an oil bath, with visible, suspended particles slowly turning to a milky slurry as the reaction progressed. At the end of this time, the reaction was quenched by pouring the contents of the flask onto 1.5 L of ice. The mixture was left defrosting overnight and was decanted the next day. The resulting suspension was then concentrated to 400 mL (ultra-filtration through 300 kDa polyethersulfone, PES, membranes) and centrifuged at 8600× *g* for 2 h. The supernatant was concentrated by ultrafiltration to minimize losses, combined with the pellets to a 100 mL volume, and dialyzed against water in 5-L beakers. After seven dialysis cycles, the suspension was sonicated three times at 130 watts, 20 kHz, and 40% amplitude for 10 min in an ice bath. The homogeneous suspension obtained was taken up to 250 mL. Then, 100 µL of CHCl_3_ were added to avoid fungal contamination and kept at 4 °C. Cellulose concentration (1%) was determined gravimetrically by drying 0.5-mL aliquots at 90 degrees in a vacuum oven until constant weight (five replicates).

After papain digestion and centrifugation, the pooled supernatants from the tunic were purified by overnight precipitation at 4 °C (3 volumes of ethanol containing 0.6 M NaOH). Alkaline hydroalcoholic precipitation at this concentration of NaOH has proved effective in removing protein [28]. The solids were separated by centrifugation (16,783× *g*, 30 min). The pellet was resuspended in ultrapure water and the alkaline hydroalcoholic precipitation was repeated. Then, the polymeric material in the resulting aqueous solution was separated in a 30-kDa Amicon Ultracentrifugal filter (Merck-Millipore), and the retentate was washed with water and finally freeze-dried.

Supernatants from the internal organs after papain digestion and centrifugation were purified by overnight precipitation at 4 °C with 0.8 volumes of ethanol containing 0.6 M NaOH. The solids were separated by centrifugation (16,783× *g*, 30 min) and the pellet was resuspended in ultrapure water. Then, the alkaline hydroalcoholic precipitation was repeated. The material in the aqueous solution was dialyzed against water (47-kDa membranes) and freeze-dried.

### 2.3. Cellulose Characterisation

The morphology of cellulose tunic membranes was analyzed by Atomic Force Microscopy (AFM) imaging. AFM was obtained with a Multimode 8 nanoscope (Veeco, NY, USA) on Peak Force Tapping Mode using a Cantilever Scanasyst-Air (tip Roc < 10 nm, silicon tip on nitrile lever, K = 0.4 N/m, frequency = 70 kHz).

The crystallinity was studied by X-ray diffraction (XRD) using an XPert Pro (PANanalytical) diffractometer. The following expression was used to determine the crystallinity index % (*CI*) [29]:

$$CI=\frac{I_{t}-I_{a}}{I_{t}}\cdot 100$$

where *I_t_* is the total intensity of the (2 0 0) peak at 22.7° 2*θ* (cellulose I) and of the (0 2 0) peak at 21.7° 2*θ* (cellulose II), and *I_a_ is* the intensity of amorphous cellulose at 18° 2*θ* (cellulose I) and at 18° 2*θ* (cellulose II).

Fiber identity was evaluated by infrared (IR) spectrometry with a Nicolet 6700 FT-IR (Thermo Scientific, MA, USA) with a KBr beam splitter, in the wavenumber interval of 400–4000 cm^−1^ at a resolution of 4 cm^−1^ and 34 sample scans. Dispersed nanocellulose was characterized by SEM microscopy over a silicon wafer surface washed in sulfuric acid for 2 h (HRSEM, Auriga Compact, ZEISS recorded at 5 kV after coating with 1 nm of platinum). One drop of 10 µL of diluted cellulose dispersion (0.0005%) was placed on the surface and left to dry overnight. The low concentration was selected to observe isolated cellulose fibers. The length, width, and aspect ratio were determined using ImageJ software from 30 individual crystals in the SEM images.

### 2.4. Polysaccharide Characterisation

Molecular weight was determined by gel permeation chromatography (GPC) on an Agilent 1260 HPLC consisting of a quaternary pump, injector, column oven, refractive index (RID), and dual-angle static light scattering (DALS) detectors. Standard and samples were dissolved in the mobile phase (0.1 M NaN_3_:0.01 M NaH_2_PO_4_, pH 6.6) and separated with four columns (PSS, Germany), Suprema precolumn (5 μm, 8 × 50 mm), Suprema 30 Å (5 μm, 8 × 300 mm), Suprema 100 Å (5 μm, 8 × 300 mm), and Suprema ultrahigh (10 μm, 8 × 300 mm), at 1 mL/min after a 100-μL injection. The column oven and DALS were kept at 30 °C and the RID at 40 °C. Detectors were calibrated with a polyethylene oxide standard (PSS, Germany) of 106 kDa (Mp) and a polydispersity index of 1.05. Refractive index increments (dn/dc) were calculated from the RID detector, assuming accurate polysaccharide concentrations for glycogen from the internal organs.

The spectra of tunic polysaccharides were measured at 25 °C on a Bruker NEO 17.6 T spectrometer (proton resonance 750 MHz), equipped with a ^1^H/^13^C/^15^N triple resonance PA-TXI probe with deuterium lock channel and shielded PFG z-gradient. The spectrometer control software was TopSpin 4.0. The chemical shifts reported are referenced to the lock deuterium solvent. Spectra were processed and analyzed with Mestrenova software v14.0 (Mestrelab Inc., Santiago de Compostela, Spain).

NMR diffusion-ordered spectroscopy (DOSY) experiments were acquired on a Bruker AVANCE DPX600. The gradient varied linearly across 64 experiments from 0.96 to 45.74 G·cm^−1^. The pulse sequence used included 2D sequence for diffusion measurement using simulated echo and LED (longitudinal-eddy-current delay), with bipolar gradient pulses for diffusion using two spoil gradients [30]. The diffusion time (Δ) was set at 200 ms and the duration of the gradients (δ) in the sequence was 1 ms.

The ^1^H Dfilter spectrum: 1D ^1^H diffusion-filtered spectra(^1^H-Dfilter) were measured with the Stimulated-Echo-LED method with bipolar pulsed field gradients (PFG, pulse sequence ledbpgp2s1d) [31]. The diffusion delay Δ was set to 100 ms (or 200 ms) and the LED period was set to 5 ms. The bipolar gradient pulses (δ) used to encode/decode diffusion had a total duration of 2 ms (or 3 ms) and were applied at the maximum gradient peak power of 50.2 G/cm. The interscan delay (d1) was set to 2 s and the number of scans was 128. The total measurement time of each ^1^H-D filter spectrum was ~10 min.

The 2D D filter-CLIP-COSY and 2D CLIP-COSY spectra: The sequence of the 2D D-filter-CLIP-COSY spectrum was implemented by a combination of an initial diffusion filter element (D-filter) based in the Bipolar gradients-STimulated-Echo (BPP-STE) [31] followed by the sequence of the CLIP-COSY [32]. The purpose of the diffusion filter is to clean the CLIP-COSY spectrum from the undesired peaks of low-molecular-weight impurities and the residual HDO solvent peak to obtain a clean spectrum of the sample under study. The D-filter used a diffusion delay Δ of 200 ms, bipolar gradient pulses (δ) of a total duration of 3 ms that were applied with the maximum gradient peak power of 50.2 G/cm. The overall duration of the perfect echo of the CLIP-COSY part was optimized for nJHH > 2 Hz corresponding to a duration of 50 ms. The center of the spectrum in F1 and F2 dimensions was set at 3.23 ppm and the spectral width in the two dimensions was 7 ppm. The interscan delay (d1) was 1.6 s, the number of scans was 32, and the number of complex points measured in the direct and indirect dimensions was 4000 and 400, respectively. The total measurement time was ~7 h 48 min. For comparison, the original CLIP-COSY spectrum without the D-filter part was measured under the same aforementioned conditions.

The 2D Dfilter-TOCSY and 2D TOCSY spectra. The sequence of the 2D D-filter-TOCSY spectrum was implemented by a combination of an initial diffusion filter element (D-filter) based on the Bipolar gradients-STimulated-Echo (BPP-STE) [31] and a version of 2D TOCSY experiment with PFG gradients (sequence “dipsi2gpphpr” of the Bruker library). The diffusion filter part had the same purpose and used the same parameters as the 2D Dfilter-CLIP-COSY spectrum described above. The TOCSY mixing time used DIPSI-2, and the duration was 80 ms. The center of the spectrum in both the F1 and F2 dimensions was set at 3.23 ppm and the spectral width in the two dimensions was 7 ppm. The interscan delay (d1) was 2 s, the number of scans was 64, and the number of complex points measured in the direct and indirect dimensions was 4k and 160, respectively. The total measurement time was ~7 h 48 min. For comparison, the analogue TOCSY spectrum without the D-filter part was measured under the same aforementioned conditions.

The 2D Dfilter-HSQC and 2D HSQC spectra. The sequence of the 2D D-filter-HSQC spectrum was implemented by the combination of an initial diffusion filter element (D-filter) based in the Bipolar gradients-STimulated-Echo (BPP-STE) [31] and a version of 2D HSQC experiment with PFG gradients (sequence “hsqcctetgpsp.2” of the Bruker library). The diffusion filter part had the same purpose and used the same parameters as the 2D Dfilter-CLIP-COSY spectrum described above. The INEPTs’ transfers of HSQC were optimized for a nominal value of a JCH of 145 Hz. The relaxation delay (d1) and the FID acquisition time (aq) were 1.6 and 0.194 s, respectively. The center of the spectrum in the ^1^H dimension (F2) was set at 3.23 ppm and the spectral width was 7 ppm. The center of the spectrum in the 13C dimension (F1) was set at 75 ppm and the spectral width was 165 ppm. The spectrum was acquired with 64 scans per t1 increment and the total measurement time was ~5 h 25 min. For comparison, the HSQC spectrum without the D-filter part was measured under the same aforementioned conditions.

The ^1^H NMR of glycogen was recorded at 25 °C on a 400-MHz Bruker Avance II. Chemical shifts are expressed in ppm with residual HOD solvent signal as a reference. Glycogen identity was confirmed by reaction with α-amylase from *Aspergillus oryzae* (Sigma-Aldrich, Prod. No. A9857, 150 U/mg protein) in 0.1 M NaAc (pH 4.7) at an enzyme-to-substrate ratio of 150 U enzyme/mg substrate. The reaction was carried out overnight at 37 °C, followed by inactivation by boiling at 95 °C for 15 min. The solution was centrifuged at 12,875× *g* for 30 min, and the supernatant was collected and filtered through 0.2-µm PES syringe filters, along with untreated glycogen (2 g/L in 0.1 M NaAc). Both solutions were injected onto the GPC system described above.

## 3. Results and Discussion

After manual cleaning of superficial incrustations on the 14 ascidian specimens (183.7 g), we separated the tunic (118.9 g) from the internal organs (64.8 g). Proximate analysis of both tissues resulted in 80.4 ± 3.2% moisture, 4.0 ± 0.3% ash, and 15.9 ± 2.9% organic matter for the tunic, and 84.4 ± 1.4% moisture, 3.2 ± 0.2% ash, 12.5 ± 1.4% organic matter for the flesh, expressed as % of wet weight ± standard deviation (*n* = 3). The tunic, therefore, accounted for the bulk of the weight of the animal (65%).

**Figure 2:**
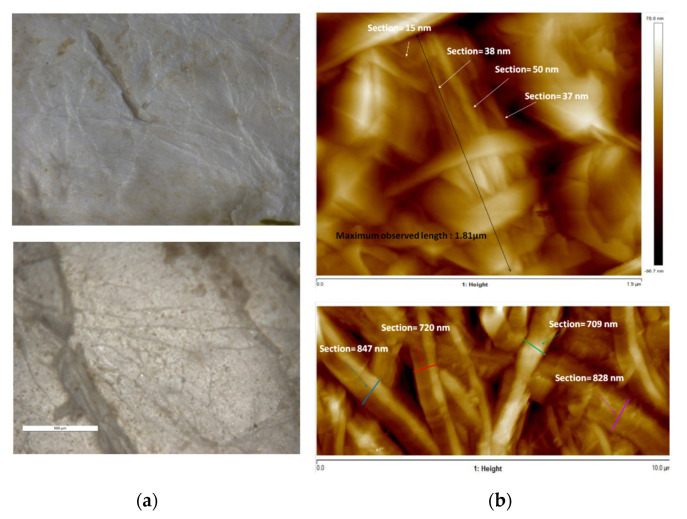
Optical microscopy images of the inner surface of native tunics (**a**) and atomic force microscopy images of the tunic interior (**b**).

Defatting, deproteinization, and bleaching of the tunics rendered a whitish, rugged material. Microscopic observation of this freeze-dried material provided an initial assessment of the native disposition of the fibers. Exfoliation of the inner surface of the tunic was required to expose the internal structure, as no fibers could be observed in the original surface. AFM measurements showed bundles of fibers close to 1 μm thick and longer than 10 μm. These bundles seemed to be formed by thinner fibers of 15–50 nm and a maximum observed length of 1.8 μm (Figure 2).

### 3.1. Nanocellulose

The sulfuric acid treatment applied to the defatted, deproteinized, and bleached tunics resulted in the disaggregation of the fiber bundles seen in AFM into individual filaments. This separation of fibers is favored by the electrostatic repulsion induced by the sulfate esters on the material surface after sulfuric acid hydrolysis [33]. While sulfuric acid is generally preferred because the strong negative charge also increases the stability of colloidal suspensions, other treatments may lead to different surface functionalities, desirable depending on the intended application [10,34,35]. For instance, carboxylation increases the binding of metals, useful in water treatment [34,35,36,37,38].

**Figure 3:**
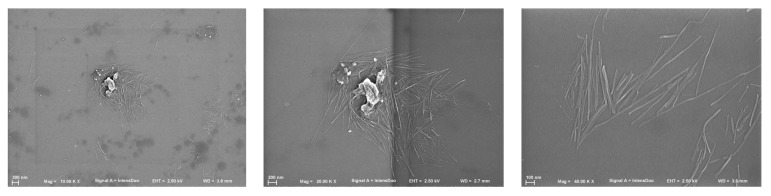
Scanning electronic microscopy (SEM) images of cellulose fibers extracted from the tunic of *Styela clava* after sulfuric acid treatment.

Scanning electron microscopy (SEM) images were performed after drying from a diluted solution. This allowed the observation of individual crystals (Figure 3) and, therefore, the determination of the length, width, and aspect ratio of 30 individual crystals. The average length was 1.2 ± 0.6 µm and the width was 19 ± 5 nm. The large standard deviation of the length corresponded to high variability, from a maximum of 2800 nm to a minimum of 470. These dimensions corresponded to an average aspect ratio of 64 ± 34.

The dimensions corresponded to nanocrystalline cellulose [39] and corroborated previous studies showing the tunicates as the source of nanocrystalline cellulose of the highest aspect ratios reported (see Table 1).

**Figure 4:**
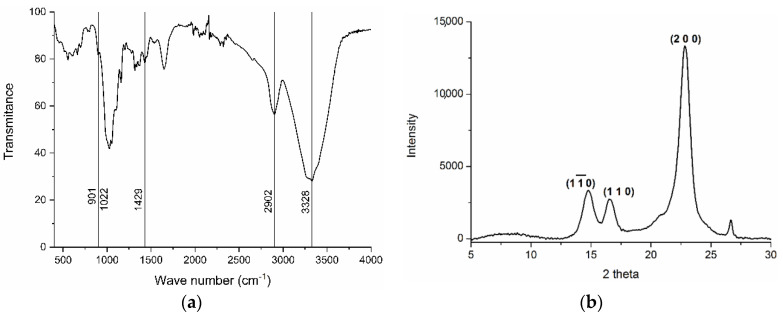
Infrared spectrum (**a**) and XRD diffractograms (**b**) of cellulose extracted from the tunic of *Styela clava*.

Infrared spectroscopy allowed identifying the isolated tunic fibers as cellulose (Figure 4a) [7,45]. The signal at 901 cm^−1^ corresponded to the glycosidic link between glucose residues. Transmission decreased around 1022 cm^−1,^ related to the C-O-C pyranose ring skeletal vibration, typically appearing at 1023–1076 cm^−1^ [45]. CH_2_ bending appeared at 1429 cm^−1^ and C-H asymmetrical and symmetrical tensile vibrations at 2902 cm^−1^. Finally, the strong signal around 3328 cm^−1^ corresponded to inter- and intra-molecular hydrogen bonds, usually in the 3050–3500 cm^−1^ region [7].

The cellulose sample analyzed by X-ray diffraction (XRD) showed the typical pattern of the crystalline form of cellulose I, as evidenced by the peaks at 2θ = 14.8°, 16.5°, and 22.8° in Figure 4b, in line with previous results [44]. Crystallinity index was calculated from the XRD diffractogram according to [29], resulting in high crystallinity at 88.6%. These results align with previous works reporting values of 89% [40] and 70% [46] in cellulose from *Styela clava*. The final 2.5 g of nanocrystalline cellulose obtained represented a 17% yield considering the dry weight of tunic, slightly higher than 12.2% previously reported in the same species [41]. This highlights the feasibility of *S. clava* as a source of nanocellulose, with improvement in yield possible through process optimization.

### 3.2. Tunic Polysaccharides

**Figure 5:**
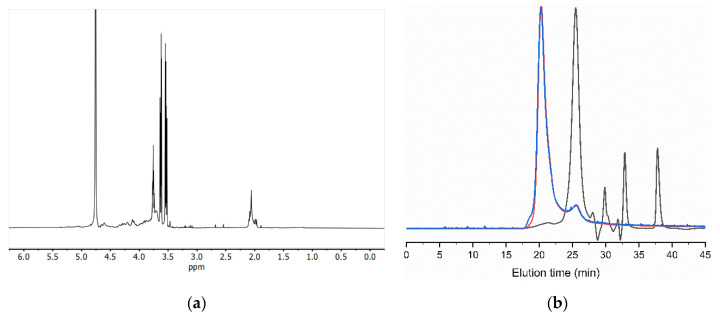
The ^1^H NMR (**a**) and GPC eluogram (**b**) of the unfractionated tunic polysaccharides. Black trace: refractive index detector (RID); red trace: right-angle light scattering detector (RALS); and blue trace: low-angle light scattering detector (LALS).

Initial characterization of the tunic isolated by ^1^H NMR suggested polysaccharide composition, with the bulk of the signals concentrated between 3.0 and 4.7 ppm (Figure 5a). Further compositional assessment of the polysaccharides by GPC revealed two polymeric peaks eluting at 20.8 and 24.9 min (Figure 5b). The first eluting peak only accounted for 7% of the refractive index detector (RI) area, with the major compound being the second one (93% of RI). The eluting time and intense light scattering signal of the first peak indicated a high-molecular-weight compound. While the unknown nature and impurity of the polymer prevented us from exactly determining its molecular weight, estimates using extreme polysaccharide refractive index increments (dn/dc) of polyanions such as chitosan (dn/dc = 0.18) [47] and chondroitin sulfate (dn/dc = 0.11) [48] resulted in Mn ranging from 3.37 to 5.51 MDa (PDI = 1.141). The same approximations applied to the second peak led to Mn from 39.7 to 65.6 kDa (PDI = 1.155).

**Figure 6:**
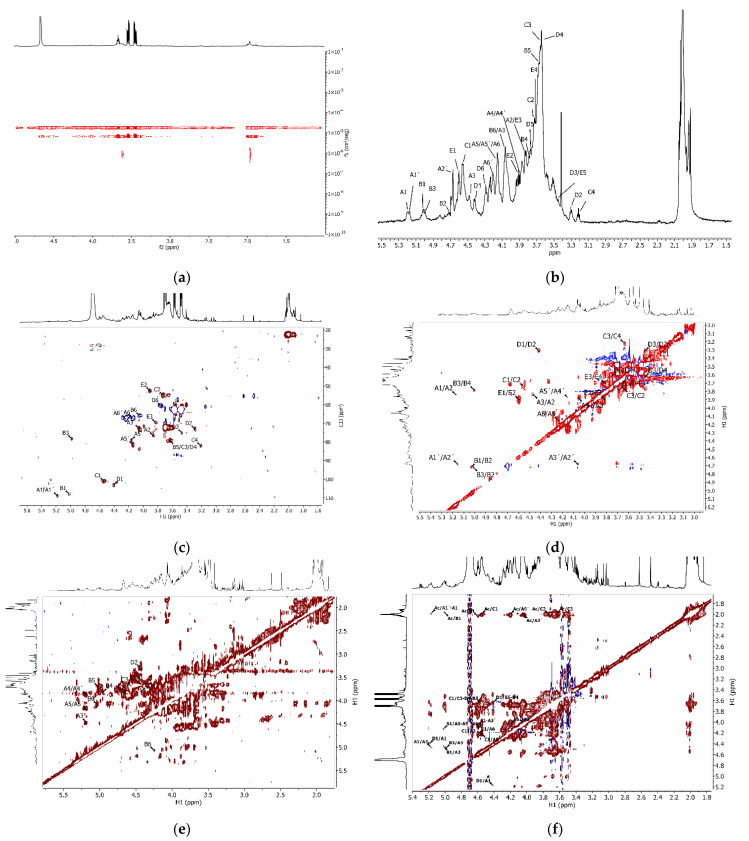
(**a**) DOSY, (**b**) ^1^H NMR (diffusion filter applied), (**c**) ^1^H-^13^C HSQC, (**d**) ^1^H-^1^H COSY, (**e**) ^1^H-^1^H TOCSY, and (**f**) ^1^H-^1^H NOESY for polysaccharides from the tunic.

Because of the limited quantity of material recovered from the tunic, we replaced conventional chromatographic fractionation by pulsed field gradients, known as NMR diffusion-ordered spectroscopy (DOSY). DOSY experiments allowed us to separate the resonances of a ^1^H NMR spectrum based on the different diffusion coefficients of the species in solution. The application of this technique to the sample from the tunic showed the presence of two macromolecules (Figure 6a), in agreement with the prior GPC analysis. Besides the band with diffusion coefficient 1.75 × 10^−5^ cm^2^·s^−1^ due to water and other low-Mw species, distinct signals appeared with diffusion coefficients of 6.59 × 10^−6^ and 9.30 × 10^−7^ cm^2^·s^−1^. Since larger molecules diffuse more slowly, the coefficients corresponded respectively to the second and first eluting compounds seen in GPC.

The bulk of the proton signals corresponded to the lower-Mw compounds, as signals for the highest-Mw species only appeared at 2.0 and 3.6 ppm. The first signal was typical of acetamide in N-acetylated sugars, while the second may have arisen from a number of polysaccharides. No further signals appeared, preventing further characterization of this minority fraction (7% of the RI area in GPC).

Taking advantage of pulsed-field gradients, it was possible to acquire a cleaner spectrum (Figure 6b) by applying diffusion filters and removing signals that did not correspond to the polysaccharides of interest (water and the intense signals at 3.51–3.56, 3.60–3.65, and 3.73–3.78 ppm) (Figure 5a). Because of the low intensity of some signals in the diffusion-filtered spectra (Figure S1, Supplementary Material), we complemented the analysis with unfiltered 2D spectra (Figure 6c–f). Signal assignment of the major sample constituents seen in GPC started by identifying the anomeric protons in the ^1^H-^13^C HSQC correlation plot (Figure 6c). Five main contours stood out at 5.18 (5.16)/108.83 (A/A’), 5.02/107.87 (B), 4.54/101.1 (C), 4.41/103.3 (D), and 4.60/101.42 (E) ppm. Then, the ^1^H-^1^H COSY spectrum allowed determining the chemical shifts of protons in the ring systems assigned to each anomeric carbon by following the correlations between neighboring protons. This procedure permitted us the assignment of protons H1–H5 in monomer A, H1–H5 in monomer D, H1–H3 in monomer A, and H1–H2 in monomer C (Figure 6d). The ^1^H-^1^H TOCSY NMR (Figure 6e) confirmed some of these assignments and revealed new correlations, as displayed in Table 2. Additional sugars, present in a low proportion, remained unidentified, illustrating the complexity of the constitutive polysaccharides.

**Figure 7:**
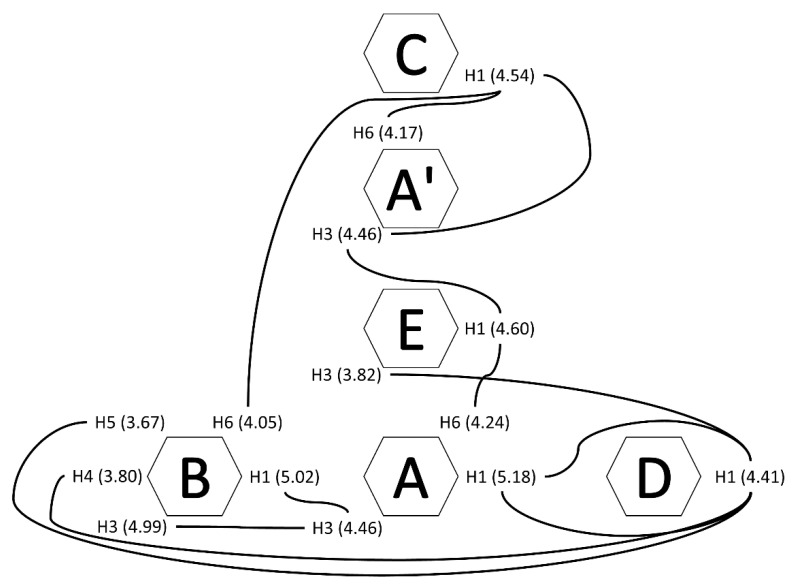
Inter-residue correlations from NOESY spectrum.

Inter-residue correlations assessed by ^1^H-^1^H NOESY NMR (Figure 6f) indicated that acetylation predominantly occurred in ring C, as shown by the cross-peaks for H1 at 4.56/2.00 ppm, H2 at 3.73/2.00 ppm, and H3 at 3.64/2.00 ppm. Weak contours also appeared at 5.17/1.99, 5.01/1.97, and 4.60/2.04 ppm, indicating partial acetylation or proximity to the highly acetylated C residue in units A/A’, B, and E. Interestingly, cross-peaks of acetyl at 2.00 ppm with signals at 4.04 and 4.21 ppm produced relatively intense contours, which may have been due to correlations of acetyl protons of C with A3’ and A6’, respectively. Additional NOESY correlations of C1 (4.54 ppm) with A3’ (4.06 ppm) and A6’ (4.21 ppm) strengthened the hypothesis that residues C and A’ were linked. Further analysis, displayed in Figure 7, allowed establishing correlations among other residues. 

The substantial overlap and the differences to previously described polysaccharides hampered the elucidation of the complete polysaccharide structure. Still, the NMR analysis can provide copious relevant structural information. For instance, chemical shifts of anomeric ^1^H in residues A (5.18 ppm), A’ (5.16 ppm), and B (5.02 ppm) pointed to α-configurations, whereas in residues C (4.54 ppm), D (4.41 ppm), and E (4.60 ppm) they indicated β-anomers (Table 1). Within this last group, residues C and E displayed similarities, remarkably the chemical shifts of C2 (55.02 and 52.44 ppm, respectively) typical of amino sugars. This agreed with N-acetylation seen in NOESY, mainly in C but to a lesser extent also in E, which may have been due to residue E being only partially acetylated. Therefore, residues C and E may resemble N-acetyl glucosamine (GlcNAc) and N-acetyl galactosamine. The (GalNAc) constitutive of glycosaminoglycans. Their chemical shifts were similar to those of GlcNAc in hyaluronic acid or GalNAc in chondroitin (Table 3). Still, they did not perfectly match them, reflecting that the described polysaccharide was related but structurally different from HA and CS. The low δ (69.52 ppm) of C3 in residue E, in particular, may indicate that this position was not substituted, as opposed to C3-links in hyaluronic acid and chondroitin.

Previous works mainly identified sulfated galactans in the tunic of ascidians, with high variability and structural heterogeneity among species [21,22,23,52]. In those cases, the entire structure of the polysaccharide (e.g., the connectivity of the residues) was not reported either. Specifically, in the structure of the ascidian from the same genre (*Styela plicata*) only low-resolution ^1^H and^13^C NMR were available, and most information was obtained from methylation analysis. Still, given the similarity of the NMR available data to the herein described, we hypothesized that residues A/A’, B, and D corresponded to variations of sulfated galactans. In fact, residue D resembled a sulfated galactan isolated from *M. exasperatus* [22] (Table 4), with C4 at 79.19 ppm suggesting substitution at this position, possibly a glycosidic link. 

Residues A/A’ were fairly similar, with overlapping H4 and C1 signals. H1 (δ 5.16/5.18) matched anomeric protons reported in sulfated galactans in other ascidians [21,22], but C1 was shifted downfield (108.83 ppm). The same occurred for residue B, with H1 similar to that of sulfated galactans in *M. exasperatus*, while the C1 signal appeared at 107.87 ppm (Table 4). Such deshielding of anomeric carbons has been reported in furanosides [53], but also in sulfated pyranosides such as sulfated iduronic acid [23]. This uronic acid has been found as part of dermatan sulfate chains in the internal organs of several species of ascidians, and a fraction could hypothetically have remained in the tunic extract. However, this appears unlikely. Although some chemical shifts of residues A/A’ and B agreed with those of iduronic acid, we saw no evidence of the acid group, which should displace downfield the C6 signals. The information gathered by NMR analysis did not permit, therefore, to determine the structure for residues A/A’ and B, but H2 signals in A’/B and H3/C3 in A indicated possible sulfation.

The polysaccharides isolated from the tunic may have been responsible for the bioactivities previously observed in extracts of *S. clava*, such as UV protection [11], prevention of hepatic injury [12], and anti-inflammatory effects [13]. Further work would be required to test the biological activity of the individual polysaccharides found.

### 3.3. Glycogen from the Internal Organs

**Figure 8:**
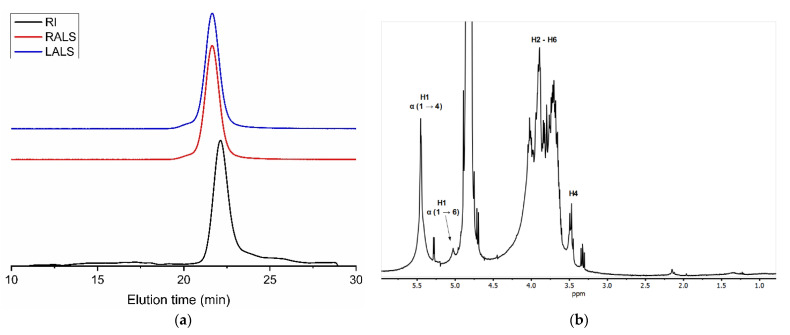
GPC eluogram (**a**) and ^1^H NMR spectrum (**b**) of glycogen extracted from the internal organs of *Styela clava*. Black trace: refractive index detector (RI); red trace: right-angle light scattering detector (RALS); and blue trace: low-angle light scattering detector (LALS).

Precipitation with 3 volumes of ethanol and 0.6 M NaOH of the liquid fraction resulting from papain hydrolysis of the internal organs of *S. clava* led to a polymeric peak in GPC eluting at 20.4 min (Figure 8a). The high intensity of light scattering signals relative to the concentration detector (refractive index) indicated a high-molecular-mass compound. This was estimated at 7.0 MDa with an associated polydispersity index of 1.879 (dn/dc = 0.135).

The ^1^H NMR spectra of the purified material showed the majority of signals overlapping between 3.4 and 4.4 ppm, typical of polysaccharides (Figure 8b). Furthermore, the strong signal at 5.45 ppm matched the characteristic chemical shift of protons bound to anomeric carbons. The lack of acetyl and methyl signals at around 2 ppm and 1 ppm suggested polymannose or polyglucose such as glycogen. Since the latter is widespread in animals, we tested glycogen first, finding good agreement with previously published NMR spectra [54]. The best resolved peaks were assigned to protons bound to anomeric carbons at 5.45 ppm (corresponding to α (1→4) glycosidic links) and at 5.03 ppm (due to α (1→6) glycosidic links), as well as protons bound to carbon 4 at the nonreducing end (3.47 ppm).

We further confirmed the identity of the polysaccharide as glycogen by reaction with α-amylase. The enzyme cleaves α (1→4) glycosidic bonds in polysaccharides containing three or more (1→4)-α-linked D-glucose units. After treatment with α-amylase, the polymeric peak eluting at 20.4 min completely disappeared.

Glycogen is a branched polysaccharide used as energy storage by animals, fungi, and bacteria. Beyond its original function, glycogen has found uses as a cosmetic ingredient [55], a precipitating agent for nucleic acids, and a potential carbon source in culture media for biotechnological applications [56]. Although glycogen isolated from *S. clava* could fulfil these applications, a low yield was obtained (3 mg/g of fresh flesh weight) compared to other sources such as mussels (10.3–66.4 mg/g) [57].

Previous works described highly sulfated dermatan sulfate and heparin-like polymers in the internal organs of other ascidian species, some of them with anticoagulant activity [23,24], but we did not find these polysaccharides in the flesh of *S. clava*.

## 4. Conclusions

Among the polysaccharides isolated from *S. clava*, the highest yield was obtained for cellulose from the tunic. Its high crystallinity and aspect ratio makes it a promising nanomaterial. Either as an invasive species or as a potential food by-product, the tunic of *S. clava* may represent a renewable and sustainable source of nanocellulose. However, the process would need to be optimized to maximize yield, and strategies for valorizing process effluents would be required to become genuinely green and economically viable.

With this aim, we surveyed polysaccharides in the liquid phase from tunic proteolysis. We found a mixture of a high-Mw polysaccharide at a low concentration (ca. 7%) and a major polysaccharide of 40–66 kDa. Detailed NMR analysis of this last fraction revealed a polysaccharide formed by at least six sugar residues. These residues include N-acetylated sugars (usually found in glycosaminoglycans) and possibly sulfated galactans. Not all residues could be identified, highlighting the need for further work to elucidate the whole structure. Furthermore, the biological activity of the isolated polysaccharides would need to be assessed to identify potential applications.

Finally, we isolated glycogen from the flesh of *S. clava*, but the low yield obtained probably hampers its utility, as other glycogen sources such as mussels are much richer. 

## Figures and Tables

**Table 1:** Characteristics of the cellulose nanocrystals from different sources compared to this work.

Source	Ref	Length (nm)	Width (nm)	Aspect Ratio
Bacterial	[39]	100–1000	10–50	20
Cotton	[39]	100–300	15	10–20
*S. clava* (tunicate)	[40,41]	1500 ± 600	18 ± 10	90 ± 30
*S. clava* (tunicate)	This work	1200 ± 600	19 ± 5	60 ± 30
*S. plicata* (tunicate)	[40]	1000–3000	20	50
*Halocinthyia roretzi* (tunicate)	[42,43]	1600	30	50
*Ciona intestinalis* (tunicate)	[40,44]	1300 ± 600	16–20	65

**Table 2:** Chemical shifts (ppm) of ^1^H and ^13^C NMR signals in tunic polysaccharides.

	A	A’	B	C	D	E
H1/C1	5.18/108.83	5.16/108.83	5.02/107.87	4.54/101.1	4.41/103.3	4.60/101.42
H2/C2	3.84/76.61	4.68/-	4.72/-	3.74/55.02	3.31 */73.07	3.91 */52.44
H3/C3	4.46 */68.18	4.06/72.74	4.99/78.22	3.65 */79.19	3.45/75.03	3.82/69.52
H4/C4	3.88 */-	3.88 */-	3.80 */-	3.22/82.09	3.63 */79.19	3.70/-
H5/C5	4.17 */78.87	4.15 */81.44	3.67 */79.19	3.48	3.76 */-	3.47/-
H6/C6	4.24 */67.4	4.17 */67.2	4.05 */67.6	-	4.27 */66.8	-
Ac	1.99	1.99	1.97	2.00		2.04

* TOCSY assignments.

**Table 3:** Chemical shifts (ppm) of ^1^H and ^13^C NMR signals of residues C and E compared to literature values. The ^1^ N-acetyl glucosamine in hyaluronic acid hexasaccharides; ^2^ N-acetyl glucosamine in hyaluronic acid from mussels; ^3^ N-acetyl galactosamine in desulfated chondroitin sulfate.

	C	E	^1^ GlcNAc [49]	^2^ GlcNAc [50]	^3^ GalNAc [51]
H1	4.54	4.60	4.55 (4.71 terminal)		4.56
H2	3.74	3.91	3.84		4.04
H3	3.65	3.82	3.71		3.88
H4	3.22	3.70	3.52		4.13
H5	3.48	3.47	3.48		3.74
H6					3.78/82
C1	101.1	101.42	103.32 (97.6 terminal)	103.52	103.8
C2	55.02	52.44	57.12	57.11	54.5
C3	79.19	69.52	85.32	85.38	78.6
C4	82.09		71.24	71.25	79.4
C5			78.13	78.5	77.5
C6			63.37		

**Table 4:** Chemical shifts (ppm) of ^1^H and ^13^C NMR signals of residues A/A’, B, and D compared to literature values for sulfated galactans isolated from the tunic of different species of ascidians (SG1: *Clavelina* sp. [21]; SG2: *H. monus* [52]; SG3: *M. exasperatus* [22]) and iduronic acid from the flesh of different species of ascidians [23] (IdoA: α-L-iduronic acid; IdoA 2S: α-L-iduronic acid sulfated on C2).

	A	A’	B	D	IdoA 2S	IdoA	SG1	SG2	SG3
H1	5.18	5.16	5.02	4.41	5.14–5.16	4.88–4.90	5.18/4.65/4.47		5.15/5.01/4.47
H2	3.84	4.68	4.72	3.31	4.14–4.17	3.51–3.54			3.87/3.83/3.31
H3	4.46	4.06	4.99	3.45	4.20–4.32	3.90–4.06			4.08/3.99/3.57
H4	3.88	3.88	3.80	3.63	4.03–4.06	4.06–4.10			4.81/4.76/3.78
H5	4.17	4.15	3.67	3.76	4.83–4.85	4.70–4.80			4.11/4.03/4.08
H6	4.24	4.17	4.05	4.27	-	-			3.70/3.75/3.39–4.11
C1	108.8	108.8	107.9	103.3	101.2–106	105.3–105.7	101.3	102.7	100.7/101.9/104.6
C2	76.6	-	-	73.1	73.8–75.2	71.6–72.0	68.6	69.9	71.9/71.7/76.0
C3	68.2	72.7	78.2	75.0	69.0–71.0	73.2–73.7	77.7	78.8	71.5/72.4/77.1
C4	-	-	-	79.2	79.2–80.7	82.3–82.7	77.1	78.8	80.9/80.8/79.6
C5	78.9	81.4	79.2	-	68.0–69.7	72.0–72.4	72.6	73.9	73.3/73.6/73.4
C6	67.4	67.2	67.6	66.8	-	-	60.7	62.2	64.0/64.4/66.3

## Supplementary Materials

The following is available online at https://www.mdpi.com/article/10.3390/polym14010016/s1. Figure S1: the 2D NMR for polysaccharides from the tunic after applying the diffusion filter. (a) ^1^H-^13^C HSQC, (b) ^1^H-^1^H COSY, and (c) ^1^H-^1^H TOCSY.
