# Supplementary material for: Isolation and Characterization of Polysaccharides from the Ascidian Styela clava
Source: Polymers (Basel). 2021 Dec 22;14(1):16. doi: 10.3390/polym14010016 (PMC8747265; doi:10.3390/polym14010016)
Supplement: Supplementary file 1 [file polymers-14-00016-s001.zip › polymers-1508280-supplementary.pdf]

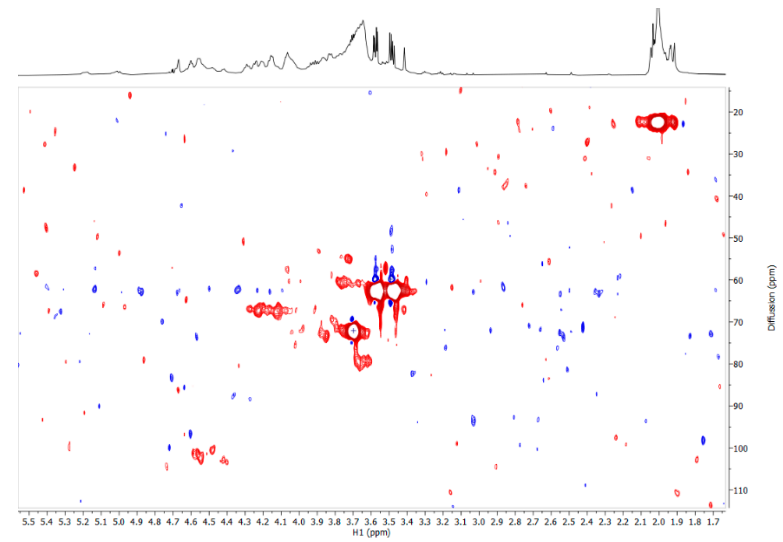

(a)

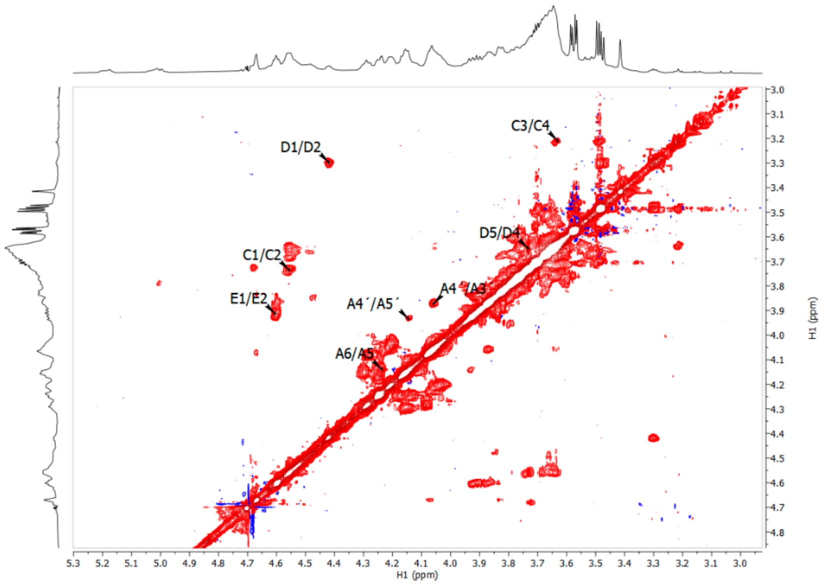

(b)

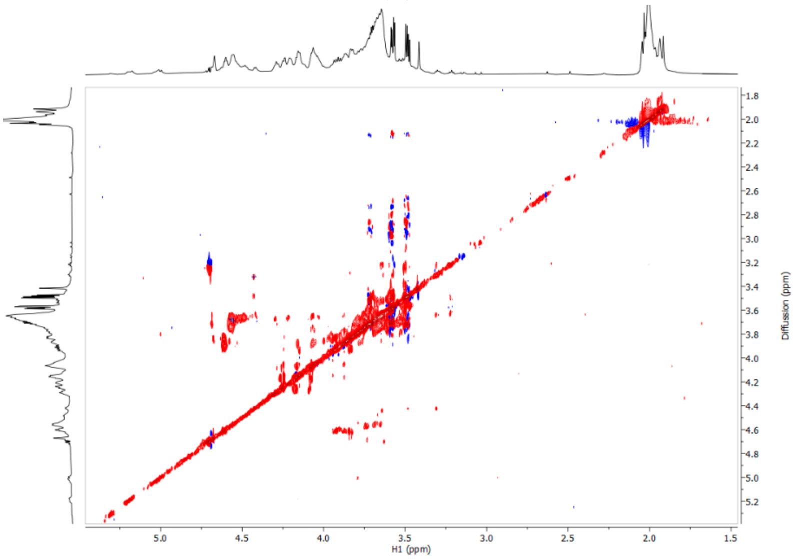

(c)

Figure S1. 2D NMR for polysaccharides from the tunic after applying the diffusion filter. **(a)** ^{1}H-^{13}C HSQC, **(b)** ^{1}H-^{1}H COSY, and **(c)** ^{1}H-^{1}H TOCSY.
